# A Geospatial Analysis of Freestanding and Hospital Emergency Department Accessibility via Public Transit

**DOI:** 10.5811/westjem.2019.3.41385

**Published:** 2019-04-16

**Authors:** Lucas C. Carlson, Olesya N. Baker, Jeremiah D. Schuur

**Affiliations:** * Brigham and Women’s Hospital, Department of Emergency Medicine, Boston, Massachusetts; † Harvard Medical School, Department of Emergency Medicine, Boston, Massachusetts; ‡ Brown University, Alpert Medical School, Department of Emergency Medicine, Providence, Rhode Island

## Abstract

**Introduction:**

Emergency departments (ED) are an important source of care for underserved populations and represent a significant part of the social safety net. In order to explore the effect of freestanding emergency departments (FSED) on access to care for urban underserved populations, we performed a geospatial analysis comparing the proximity of FSEDs and hospital EDs to public transit lines in three United States (U.S.) metropolitan areas: Houston, Denver, and Cleveland.

**Methods:**

We used publicly available U.S. Census data, public transportation maps obtained from regional transit authorities, and geocoded FSED and hospital ED locations. Euclidean distance from each FSED and hospital ED to the nearest public transit line was calculated in ArcGIS. We calculated the odds ratio (OR) of an FSED, relative to a hospital ED, being located within 0.5 miles (mi) of a public transit line using logistic regression, adjusting for population density and median household income and with error clustered at the metropolitan statistical area (MSA) level.

**Results:**

The median distance from FSEDs to public transit lines was significantly greater than from hospital EDs across all three markets. In Houston, Denver, and Cleveland, the median distance between FSEDs and public transit lines was greater than from hospital EDs by 1.0 mi, 0.2 mi, and 1.6 mi, respectively. The OR of a public transit line being located within 0.5 mi of an FSED, as compared with a hospital ED, across all three MSAs was 0.21 (95% confidence interval [CI], 0.13–0.34) unadjusted and 0.20 (95% CI, 0.11–0.40) adjusted for population density and median household income.

**Conclusion:**

In comparison with hospital EDs, FSEDs are located farther from public transit lines and are less likely to be within walking distance of public transportation. These findings suggest that FSEDs are unlikely to directly increase access to care for patients without private means of transportation. Further research is necessary to explore both the direct and indirect impact of FSEDs on access to care, potentially through effects on hospital ED crowding and overall healthcare expenditures, as well as the ultimate role and responsibility of FSEDs in improving access to care for underserved populations.

## INTRODUCTION

Since 2009 the number of freestanding emergency departments (FSED) in the United States (U.S.) has increased more than fourfold,^1^ with over 400 facilities currently operating across the country. This growth has taken place primarily in large urban areas, especially in Texas, Colorado, and Ohio.^2^ Recently, policymakers have begun to question the impact of FSEDs on access to care for underserved populations.^3^ Although FSEDs have the potential to meet the growing demand for acute unscheduled care,^3^ prior studies have demonstrated that FSEDs are preferentially located in socioeconomically advantaged areas,^2^ and so it is unclear whether expansion of FSEDs will improve access to care for the underserved.

While 15% of patients use ambulances and emergency medical services to access emergency care, an overwhelming majority of patients rely on independent means of transportation to reach the ED.^4^ For low-income populations in urban areas who often rely solely on public transportation,^5^ location of healthcare services in close proximity to public transportation is an important factor in access. To assess the potential effect of the growth of FSEDs on access to care for urban, underserved populations, we performed a geospatial analysis comparing the proximity of public bus, light rail, and metro lines to FSEDs and hospital EDs in three metropolitan areas across the U.S.

## METHODS

### Data Sources

We collected data for this analysis from multiple sources and combined them using the geographic information system (GIS) software package, ArcMap 10.1 (Environmental Systems Research Institute, Redlands, California). We obtained hospital ED addresses from the 2013 American Hospital Association database and FSED addresses from state departments of health as well as through a comprehensive systematic online search of “freestanding” or “satellite” EDs, as described elsewhere.^2^ We geocoded hospital ED and FSED addresses using the U.S. Census Geocoder.^6^ Addresses that could not be geocoded through this system were manually geocoded using Google Maps (Google Maps, Mountain View, California). We obtained values for population density and median household income for each census tract from 2010 U.S. Census data.^6^

We selected Houston, Denver, and Cleveland for inclusion in our analysis, as they had a high density of FSEDs as well as publicly available transit geodata. We defined the total study area for each city using metropolitan statistical areas (MSA), which are used by the U.S. Census Bureau to demarcate greater metropolitan areas for statistical purposes (Houston-The Woodlands-Sugar Land; Denver-Aurora-Lakewood; and Cleveland-Akron-Canton).^6^ We contacted regional transit authorities in each metropolitan area and the surrounding regions to obtain the most current available public transit route data. We chose route line data for this analysis over bus and metro stop point data because up-to-date stop data were not available across all three MSAs and the use of line data avoided any potential confounding from stop density along a single route.

### Data Analysis

Maps were projected in the respective state plane coordinate systems for each MSA. Using these maps, we calculated the shortest Euclidean distance from each FSED and hospital ED to a public transit line. We also calculated the number of public transit lines within a 0.25 mile (mi) and 0.5 mi radius of each FSED and hospital ED. We selected 0.25 mi and 0.5 mi as reasonable walking distances. These data along with population density and median household income of the census tract in which each ED was located were extracted from the GIS database for further analysis.

To compare the likelihood of FSEDs and hospital EDs being located within walking distance of a public transit line, we used logistic regression to calculate the odds of an FSED being located within 0.5 mi of a public transit line, relative to hospital EDs. We additionally adjusted our model for population density and median household income to account for potential confounding between public transit proximity and population density and socioeconomic factors. Odds ratios (OR) were calculated for all MSAs together with error clustered at the MSA level. As this study was an analysis of publicly available data not including human subjects, it was exempt from institutional review board approval.

## RESULTS

The median distance to public transit lines was greater for FSEDs than hospital EDs across all three MSAs (see Table 1). The difference between median distances from FSEDs and hospital EDs to public transit lines was greatest in Cleveland, with 1.6 mi ([interquartile range {IQR}, 0.4 – 6.2] for FSEDs compared with < 0.1 mi [IQR, 0.0–7.0] for hospital EDs). This difference was smallest in Denver with FSEDs having a median distance of 0.2 mi (IQR, 0.0–0.4) to public transit lines compared with < 0.1 mi (IQR, 0.0–0.1) for hospital EDs.

The median number of public transit lines within a 0.25 mi radius of FSEDs was 0 for both Houston and Cleveland. For hospital EDs, the median number of public transit lines within a 0.25 mi radius was 1.5 (IQR, 0.0–1.5), 2.5 (0.0–7.0), and 2 (1.0–4.0) in Houston, Denver, and Cleveland, respectively. Similar patterns were seen within a 0.5 mi radius, with the median ranging from 0 to 2 for FSEDs and 2 to 7 for hospital EDs across the three MSAs. These patterns are further depicted in the Figure.

The unadjusted OR of a public transit line being located within 0.5 mi of an FSED compared to an hospital ED was 0.21 (95% confidence interval [CI], 0.13–0.34); and the OR adjusted for median household income and population density was 0.20 (95% CI, 0.11–0.40). See Table 2.

## DISCUSSION

The role and responsibility of FSEDs in improving access to care for the underserved is the subject of active debate. Many independent FSEDs, operated by non-hospital, for-profit entities, are not recognized by the Center for Medicare and Medicaid Services; thus, they do not accept Medicare or Medicaid and are otherwise cost-prohibitive for most low-income individuals.^1^,^3^ Policymakers have cited concerns regarding FSEDs’ ability to improve care for the underserved and their lack of commitment to these communities.^3^ Conversely, however, the rapid expansion and uptake of their services continues to demonstrate the substantial demand for FSED services in the healthcare market.

While further research and dialogue are necessary to determine the ultimate responsibility of FSEDs to underserved populations, the findings of our study support claims that FSEDs have limited potential to directly increase access to care for urban underserved populations based on their current locations. In addition to being located nearer to patient populations with relatively higher socioeconomic status,^2^ our analysis showed that FSEDs located farther from public transit lines than hospital EDs are less likely to be within walking distance of public transportation, and are therefore less accessible for individuals without access to private means of transportation. As transportation represents a crucial barrier to care for low-income groups,^8^,^9^ it is therefore less likely that FSEDs will directly improve access to acute unscheduled care for urban underserved populations.^5^ Still, the effect of FSEDs on hospital ED crowding, wait times, and overall healthcare costs, and the potential indirect impact of these effects on access to care for underserved populations, has yet to be studied and further research is necessary to evaluate these considerations.

In prior analyses in Texas, Colorado and Ohio, FSEDs were shown to be located in areas with higher population growth, higher incomes, greater private insurance coverage, lower Medicaid prevalence, and more hospital EDs.^2^ The differences in FSED and hospital ED proximity to public transportation are reflective of active choices made, primarily, by FSEDs. Developers of FSEDs likely have multiple motivations for selecting a particular location, including population density or growth, a well-reimbursing payer mix, and lack of competing services. Proximity to public transit lines correlates with higher proportions of low-income families and, consequently, those who are uninsured or dependent on Medicaid and Medicare.^5^ It also reflects location accessibility for similar populations reliant on public transportation. The choice by FSED developers not to locate near transit routes could be an active decision, in order to avoid certain types of patients, or it could also reflect another confounding location decision, such as a preference to locate in new commercial developments. Further study will be necessary to assess the implications of these location decisions on healthcare delivery and local health systems.

## LIMITATIONS

The findings of this analysis must be interpreted in the context of several limitations. First, we used Euclidean distances. Although these are potentially less precise than walking distances, prior research has demonstrated Euclidean distances to be highly correlated with travel distance while being more practical for geographic studies.^10^ Next, FSEDs are rapidly expanding and, as of yet, there is no national registry of FSEDs. Our findings are based on a rigorous, multifaceted search strategy, but given that this is a rapidly evolving market, it is probable that new FSEDs have been constructed and some of those included have been closed since completing this search. Additionally, smaller suburban public transit lines that are not managed by regional transit authorities may have also been overlooked by our methods. Our analysis also did not account for patients who use other means of transportation to EDs, such as taxis, bicycles, or walking. Lastly, there may be other geospatial factors affecting ED location that were not included in this analysis, such as local healthcare policy, traffic patterns, or physical terrain.

## CONCLUSION

The success of FSEDs in the free market continues to demonstrate the demand for FSED services by the general public, but their potential value to urban underserved populations is limited by their present locations and accessibility by public transportation. Further research should aim to evaluate the effects of FSEDs on ED crowding, population health, and healthcare costs, as well as their indirect impact on access to care for underserved populations. Policy makers must also continue to define what obligation FSEDs ultimately have to underserved populations to guide regulatory efforts for this expanding model of emergency care delivery.

## Figures and Tables

**Figure:**
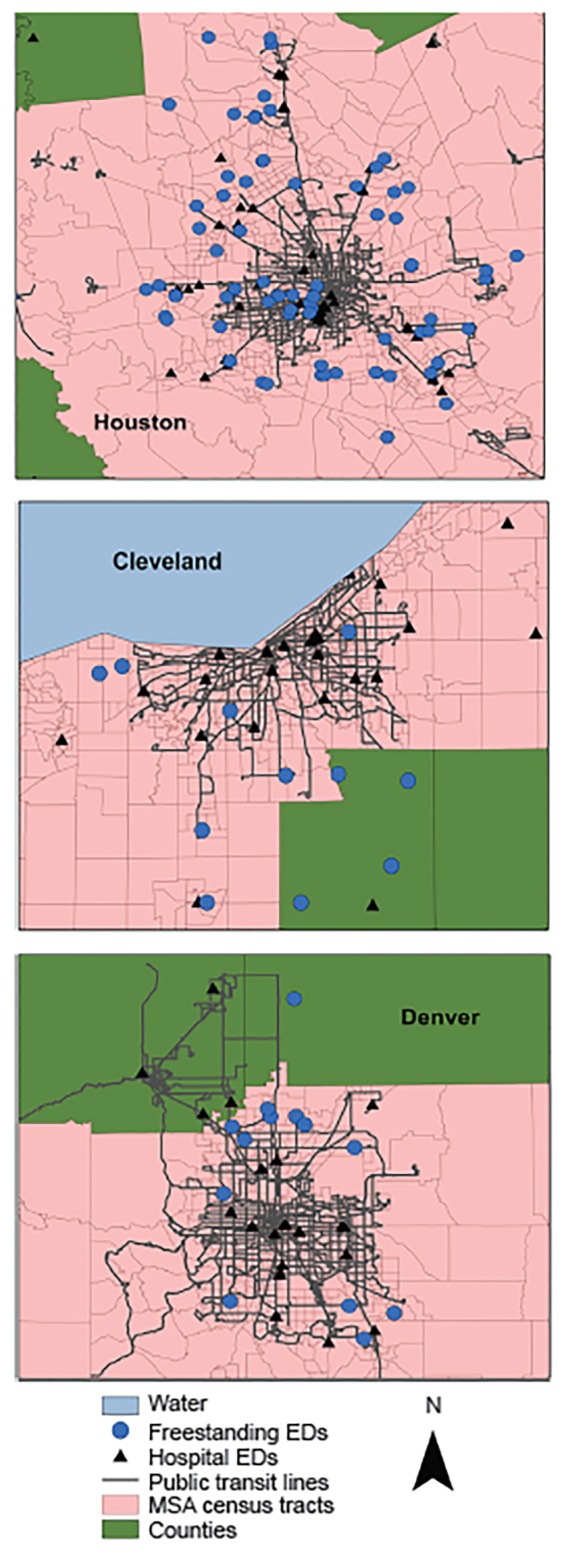
Freestanding emergency departments and hospital emergency department (ED) locations in relation to public transit routes. *MSA*, Metropolitan Statistical Area.

**Table 1:** Proximity of freestanding emergency departments and hospital emergency departments to public transit lines.

	Houston		Cleveland		Denver	
			
	FSED (N=78)	HED (N=68)	FSED (N=9)	HED (N=26)	FSED (N=12)	HED (N=19)
Distance to transit line (mi)	1.1 [0.0; 3.6]	0.1 [0.0; 0.3]	1.6 [0.4; 6.2]	<0.1 [0.0; 7.0]	0.2 [0.0; 0.4]	<0.1 [0.0; 0.1]
No. lines within 0.25 mi radius	0 [0.0; 1.0]	1.5 [0.0; 5.5]	0 [0.0; 0.0]	2.5 [0.0; 7.0]	1 [0.0; 2.0]	2 [1.0; 4.0]
No. lines within 0.5 mi radius	0 [0.0; 1.0]	2 [1.0; 12.0]	0 [0.0; 1.0]	7 [0.0; 17.0]	2 [1.0; 2.0]	4 [2.0; 6.0]

*FSED*, freestanding emergency department; *HED*, hospital emergency department; *ED*, emergency department; *No*., number; *mi*, miles.

Median, [interquartile ratio].

**Table 2:** Logistic regression of the likelihood of freestanding emergency departments being located within 0.5 miles of public transit relative to hospital emergency departments.

	Unadjusted OR	Adjusted OR
Public transit line located within 0.5 miles	0.20 [0.13 – 0.34]	0.20 [0.11 – 0.40]
Population density (in 1,000s)		1.18 [1.08 – 1.28]
Median household income (in 1,000s)		1.02 [1.02 – 1.03]

*OR*, odds ratio; *CI*, confidence interval; *FSED*, freestanding; *ED*, emergency department.

OR [95% CI].
